# Current News

**Published:** 1896-04

**Authors:** 


### Current News.

### NORTHEASTERN DENTAL ASSOCIATION.

At the Union Meeting of the Connecticut Valley and New England Dental Societies, held October, 1895, in Worcester, Massachusetts, the question of consolidation was an important one.

Committees from the two societies had been appointed the year previous looking towards that result. These committees made a favorable report, and presented a constitution and by-laws. Each society voted in favor of consolidation, and united in adopting the new constitution and by-laws, thus forming a new society of upward of two hundred and fifty members, under the name of the "Northeastern Dental Association." The following were the officers elected for the ensuing year:

President, Dr. James McManus, Hartford, Conn.; First Vice-President, Dr. James H. Daly, Boston, Mass.; Second Vice-President, Dr. C. C. Barker, Meriden, Conn.; Secretary, Dr. Edgar O. Kinsman, Cambridge, Mass.; Assistant Secretary, Dr. A. J. Cutting, Southington, Conn.; Treasurer, Dr. George A. Young, Concord, N. H.; Editor, Dr. C. W. Strang, Bridgeport, Conn.; Librarian, Dr. George F. Cheney, St. Johnsbury, Vt.

The Executive Committee and Section Committee are appointed by the president, who selected the following:

*Executive Committee.*—Dr. George A. Maxfield, chairman, Holyoke, Mass.; Dr. George L. Parmele, Hartford, Conn.; Dr. George F. Cheney, St. Johnsbury, Vt.; Dr. W. R. Blackstone, Manchester, N. H.; Dr. S. G. Stevens, Boston, Mass.

*Section Committee.*—Section I., Dr. C. T. Stockwell, Springfield, Mass.; Section II., Dr. C. A. Brackett, Newport, R. I.; Section III., Dr. William Jarvis, Claremont, N. H.; Section IV., Dr. Thomas Fillebrown, Boston, Mass.; Section V., Dr. E. S. Gaylord, New Haven, Conn.

The new society starts out harmoniously, and promises to be an important factor in dental circles in New England, and will doubtless shed its influence into the surrounding States.

EDGAR O. KINSMAN,
*Secretary.*

---

### WOMAN'S DENTAL ASSOCIATION.

THE Fourth Annual Meeting was held at the office of Dr. Mary H. Stilwell, 1718 Walnut Street, March 7, 1896. President, Dr. Anna T. Focht, in the chair.

Election of officers resulted in the following:

President, Dr. Elizabeth Davis; Vice-President, Dr. Maria T. Lasser; Recording Secretary, Dr. Hannah M. Miller; Corresponding Secretary, Dr. Bertha Jarrett; Treasurer, Dr. Emily W. Wyeth.

*Executive Committee.*—Drs. Eliza Yerkes, Anna L. Leaming, Emily W. Wyeth, Martha B. Corkhill, and Mary H. Stilwell.

Vice-Presidents for the various States represented are: Massachusetts, Dr. Mary Gallup; Rhode Island, Dr. Jennie H. Gallup; New York, Dr. Alice I. Ireland; Maryland, Dr. Fannie E. Hoopes; District of Columbia, Dr. Edith Jewell; Illinois, Dr. Hester Baker; Missouri, Dr. Alice A. Graham; Nebraska, Dr. Cora G. Little; Wyoming, Dr. Sarah Gardiner; Colorado, Dr. Sara May Townsend.

Present membership, thirty-two.

EMILY W. WYETH,
*Recording Secretary.*

3920 FAIRMOUNT AVENUE, PHILADELPHIA.

---

### HARVARD ODONTOLOGICAL SOCIETY.

AT the annual meeting of the above society the following officers were elected:

President, Waldo E. Boardman, D.M.D.; Recording Secretary, Jos. T. Paul, D.M.D.

The treasurer, editor, and corresponding secretary were re-elected.

*Executive Committee.*—Jos. T. Paul, D.M.D.; Frank T. Taylor, D.M.D.; William P. Cooke, D.M.D.

*Committee on Clinics.*—J. G. W. Wemer, D.M.D.; William P. Cooke, D.M.D.; Arthur H. Stoddard, D.M.D.

Respectfully yours,
E. B. HITCHCOCK,
*Corresponding Secretary.*

